# The relationship between low back pain and leisure time physical activity in a working population of cleaners - a study with weekly follow-ups for 1 year

**DOI:** 10.1186/1471-2474-13-28

**Published:** 2012-02-22

**Authors:** Tobias Jespersen, Marie B Jørgensen, Jørgen V Hansen, Andreas Holtermann, Karen Søgaard

**Affiliations:** 1 National Research Centre for the Working Environment, Lersø Parkalle 105, DK 2100 Copenhagen Ø, Denmark; 2 Institute of Sports Science and Clinical Biomechanics, University of Southern Denmark, Campusvej 55, DK 5230 Odense M, Denmark

## Abstract

**Background:**

Low back pain (LBP) and leisure time physical activity (LTPA) are considered to be closely related, and clinical guidelines for the treatment of acute LBP recommend patients stay physically active. However, the documentation for this recommendation is sparse and based on studies involving patient populations. The purpose of the study was (1) to investigate the correlation between LBP and LTPA on a weekly basis over the course of a year in a high-risk group of cleaners; and (2) to investigate if maintaining LTPA during an episode of acute LBP has a positive effect on LBP intensity in the subsequent 4 weeks.

**Methods:**

188 cleaners consented to participate in a 52-week text message survey about hours of LTPA and intensity of LBP (from 0 to 9) over the previous 7 days. The correlation between LBP and LTPA was calculated by Pearson correlation coefficient. During an episode of acute LBP, a mixed effect logistic regression model was used to investigate whether cleaners who maintain LTPA have a lower pain intensity and higher probability of returning to initial pain intensity within the following four weeks compared with cleaners who decrease LTPA during acute LBP.

**Results:**

The correlation between weekly LTPA and LBP data was negative, but numerically low (r = -0.069) and statistically insignificant (*p *= 0.08). Among the 82 cleaners experiencing at least one episode of acute LBP, those maintaining LTPA during an episode of acute LBP did not have a lower pain intensity (average LBP intensity difference between groups of 0.06; 95% confidence interval (95% CI) of -0.417 to 0.539) or higher probability of returning to initial pain level (Odds ratio 1,02; 95% CI of 0.50 to 2.09) in the following four weeks compared with cleaners decreasing LTPA during acute LBP.

**Conclusions:**

Hours of LTPA and intensity of LBP measured on a weekly basis throughout a year showed no close correlation. Maintaining LTPA during an episode of acute LBP did not result in a positive effect on LBP in the following 4 weeks. Documentation of LTPA recommendations for acute LBP in working populations is still needed.

## Background

Low back pain (LBP) is a common condition in the general population, but in particular is highly associated with occupational groups with low education and income and high physical work demands [1,2]. Occupational cleaning is one of the groups that fits this category and it is also among the sectors with the highest prevalence of LBP [1,3]. The cleaning industry employs approximately 3.6 million workers in the EU and LBP in this working population constitutes a considerable economic burden with high direct and indirect costs [1,2,4].

Risk factors for developing LBP are numerous, including individual, behavioral, psychosocial and work-related factors [5]. Clinical diagnostic measures related to LBP include hernia nuclei pulposi, infection, osteoporosis, rheumatoid arthritis, fracture, and tumor. However, up to 90% of LBP cases are of unknown origin [6].

LBP is usually divided into acute LBP (i.e. persisting for less than 6 weeks), sub-acute LBP (i.e. persisting for between 6 and12 weeks) and chronic LBP (i.e. persisting for more than 12 weeks) [5]. However, although the natural course of LBP is argued to be recurrent rather than acute or chronic [7], studies investigating the natural course of LBP over a longer period in a non patient group are lacking.

For several decades, LBP has been considered to be closely related to the level of physical activity. Up to the 1990s, the general recommendation for treating acute LBP consisted of restricted physical activity and bed rest [8,9]. However, research in the 1980s and 1990s revealed that a decreased physical activity level (disuse) could lead to physical deconditioning of body structures and functions, increasing the risk for acute LBP to develop into a chronic condition [10-12]. Therefore, the current clinical guidelines recommend that people with acute LBP should remain physically active and continue normal activities [5,8,9]. However, the clinical guidelines are based on few studies, primarily involving patients on sick leave [5,8,9,13,14], even though most people with acute LBP do not seek health care [15]. Currently, it is unknown if the guidelines also apply to occupationally active populations.

Studies investigating the relationship between LBP and physical activity have been cross-sectional or retrospective [10,16]. Consequently, time-dependent information on the relationship between LBP and physical activity over a longer period of time is lacking, and it is unknown if staying physically active during acute LBP actually reduces subsequent LBP in a working population. The recent increased use of mobile telephones offers a new, simple and convenient method for collecting high time frequency data on self-reported pain status and physical activity by means of Short Message Service (text messaging). Numerous studies have already used this method for different purposes [17-19]. However, to our knowledge, this is the first study to use it for providing data on LBP and physical activity in a working population, such as cleaners.

The purpose of the current study was in an occupationally active population to investigate the correlation between LBP and leisure time physical activity (LTPA) on a weekly basis throughout a year, and further, to investigate if maintaining LTPA during an episode of acute LBP has a positive effect on LBP intensity in the subsequent 4 weeks. More specifically, two hypotheses were tested (1) LBP and LTPA are negatively correlated, and (2) maintaining the usual level of physical activity during an episode of acute LBP will have a positive effect on the intensity of LBP in the subsequent period.

## Methods

### Study population

The study population were cleaners employed at six Danish workplaces, who participated in a clinical study described elsewhere [3]. Ethics approval was received from the local ethics committee (H-C-2007-0033) and the clinical trial was registered with a unique trial registration number (ISRCTN96241850).

The 238 participants were invited to take part in the current text message survey. The only inclusion criteria were that they had a cell phone and were employed for a minimum of > 20 hours per week as cleaners. Participants in the text message survey used their own cell phones and were financially compensated for the cost of the text message responses.

### Outcomes

Prior to the text message survey, all participants filled out a screening questionnaire, from which data were collected regarding age, gender, weight, height, job seniority, ethnicity, LTPA during the previous year [20] and musculoskeletal pain during the previous year according to the Standardised Nordic questionnaires for the Analysis of Musculoskeletal Symptoms, including a body chart defining the different body regions [21].

#### Low back pain

The first question in the text message survey was based on the Standardised Nordic questionnaires for the Analysis of Musculoskeletal Symptoms [21], and worded: "*On a scale from 0-9, how much pain have you experienced in the lower back region during the last week? (0 = no pain, 9 = worst pain imaginable)*".

#### Leisure time physical activity

The second question in the text message survey was based on a modified version of the International Physical Activity Questionnaire (IPAQ) [22], and was phrased: "*During the last 7 days, how many hours did you do physical activities in your leisure time?*" The participants were instructed to respond with a number for the total time in hours spent on physical activities in leisure time such as aerobics, running, bicycling, swimming and active transport to and from work in the previous 7 days.

### Procedure for collection of text message data

Hours per week of LTPA and weekly LBP intensity over the course of one year were collected from the participants through a text message survey (using SMS-Track (New Agenda Solution) [23]). The method has previously been used in other studies to measure LBP [17-19]. The definitions of LBP and LTPA were given at an information meeting prior to the initiation of the text message survey. At the meeting, the participants were also shown the pain chart in the Nordic questionnaire giving the definition of body regions. The questions were sent automatically in the form of text messages each Wednesday for 1 year (52 weeks in total). One text message was sent for each of two questions, and the participants responded to each question by returning a text message. The replies were incorporated into a data file on a server. If a participant did not respond to a text message, it was automatically sent again the following Sunday. After 2 consecutive weeks without text message responses, a reminder text message was sent: "Dear... We haven't received any answers from you to the text messages for 2 weeks. We therefore want to make sure that you have received the messages. If not, you can let us know by calling this number.... Best regards". If the participant still did not respond, a person from the research group telephoned the participant and asked the participant about the reason for the lack of response.

The text message survey was conducted from October 2008 to January 2010.

### Data analyses

#### Correlation between leisure time physical activity and low back pain intensity

A Pearson correlation coefficient was calculated for hours per week of LTPA and weekly LBP intensity for each individual, who responded by text messaging to more than 80% of the questions over the 52 weeks (high responders). The common mean correlation and confidence interval were calculated from the average and standard deviation of the individual Pearson correlations for all high responders. Furthermore, to examine time-dependent relations between the weekly amount of LTPA and LBP intensity, cross correlations were calculated for each individual, yielding estimates and confidence intervals for the common mean cross correlations based on the averages and standard deviations of the individual cross correlations for the high responders.

#### Acute low back pain episode and leisure time physical activity

**Figure 1:**
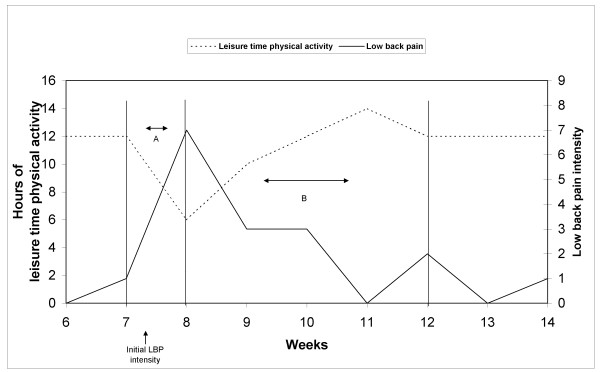
**A model illustration of hours per week of LTPA and weekly LBP intensity over 9 weeks**. An increase in LBP intensity by 2 or more from one week to the next is defined as an acute LBP episode (Period A). The following 4 weeks after the acute LBP episode is defined as the follow-up period (Period B). Initial LBP intensity is the pain intensity before the acute LBP episode.

To examine if maintaining the physical activity level in leisure time reduces acute LBP among a population of cleaners, we first defined an acute LBP episode. As no clear definition of an episode of acute LBP exists [24], we chose to define it as an increase of 2 points or more from one week to the next (Figure 1, period A) on a 10-point scale of LBP intensity. A change of 2 points or more is described in the literature as a clinically relevant change in LBP intensity [25,26].

Follow-up was defined as a time period of four weeks after the LBP episode (Figure 1, period B), since decline in acute LBP is thought to primarily occur within this timeframe [15,27]. Initial LBP intensity was defined as the pain intensity in the week preceding the incident of acute LBP (Figure 1, Week 7). Hours of LTPA in the same time period as the acute LBP episode (Figure 1, period A), as well as changes in LTPA in the same period (Figure 1, period A), were extracted for each acute LBP episode. If LTPA was reduced by 1 hour or more during the acute LBP episode, then LTPA was defined as 'decreased', otherwise it was defined as 'maintained'.

This threshold was made based on intervention studies showing effects on musculoskeletal pain from 1 hour or less of LTPA among persons with pain at baseline [28,29].

We then tested if episodes of acute LBP with maintained hours of LTPA had a higher probability of returning to initial LBP intensity during the 4-week follow-up period compared with episodes of acute LBP with decreased hours of LTPA. Also, we tested if episodes of acute LBP with maintained hours of LTPA had a lower mean increase in LBP intensity from the week preceding the episode of LBP to the 4-week follow-up compared with episodes of acute LBP with decreased hours of LTPA.

All acute LBP episodes with at least one text message response on LBP in the 4-week follow-up period were included in the analysis. Increases in LBP intensity that occurred within the 4 week period following an acute LBP episode were not considered to be new episodes. A new LBP episode could only occur if an increase in pain intensity occurred > 4 weeks after the previous LBP episode.

A mixed effects logistic regression model was used to estimate the probability of returning to the initial pain intensity within the 4-week follow-up period and to test if the probability after an acute LBP episode with decreased hours of LTPA differed from the probability after a LBP episode with maintained LTPA.

Moreover, a linear mixed effect model was used to test for difference in mean LBP intensity in the 4-week follow-up period compared with the initial pain intensity between LBP episodes with decreased hours of LTPA and LBP episodes with maintained LTPA.

In the regression models, participants were included as a random effect to account for the possibility of correlated repeated measures among the participants. The regression models were estimated in a two-step procedure. As this study was nested in a clinical study, in the first step we adjusted for allocation in the clinical study, and in the second step we adjusted for allocation in the clinical study as well as for age and gender. Data were analysed using the GLIMMIX and MIXED procedures of SAS 9.2.

## Results

### Study population characteristics

**Figure 2:**
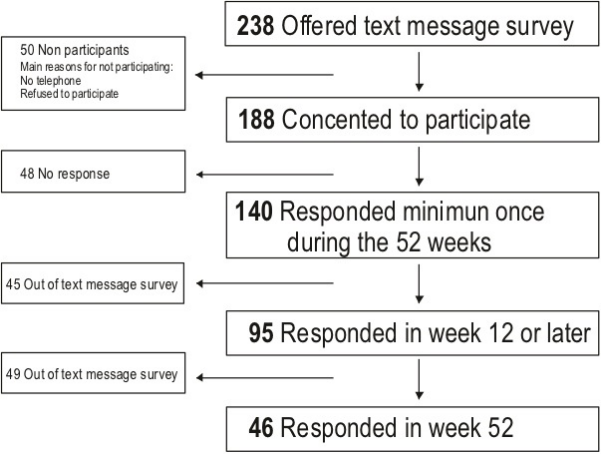
**Flow chart on text message survey participation**. Participation in text message survey was offered to 238 cleaners and 188 consented to participate. Among the 188, 48 never responded, leaving 140 participants, who responded to both questions at least once and 95 still responded in week 12. In the last week (week 52) 46 participants still responded.

Among the 238 cleaners from the six workplaces, 188 consented to participate in the text message survey. Of the 188 participants contacted in the text message survey, 140 responded by text messages for 1 week or more (Figure 2). By week 12 or later, 95 cleaners responded and by the end of the survey period (Week 52), 46 cleaners responded.

Forty-nine cleaners were high responders with a response to both text messages in more than 80% of the weeks. Participants who responded to the questions in less than 80% of the weeks were defined as low responders.

The mean age of the 238 cleaners who were offered text message participation was 44.8 years (SD 9.0) and most were female (81.5%). Mean Body Mass Index (BMI) was 26.7 (SD 4.7) and they had a mean job seniority in the cleaning industry of 8.4 years (SD 8.4).

High responders were significantly older, had longer job seniority, and a higher proportion were born in Denmark than low responders (Table 1).

**Table 1:** Heading: Baseline characteristics of high and low responders in the text message survey

		Offered text message survey (n = 238)	High responders (n = 49)	Low responders (n = 139)
Age (years)	Mean (SD)	44.4 (9.0)	47.7 (9.4)	43.2* (8.9)

Sex (females)	%	81.5	85.7	82.7

BMI (kg/m^2^)	Mean (SD)	26.7 (4.7)	26.7 (4.6)	26.5 (5.2)

Job seniority (years)	Mean (SD)	8.4 (8.4)	12.1 (10.1)	7.1* (7.1)

Last year LTPA (1-4)	Mean (SD)	2.0 (0.8)	2.2 (0.8)	2.0 (0.8)

Ethnicity (Danish)	%	48.7	83.7	42.4*

LBP (> 30 days)	%	34.2	27.7	34.6

Table legend: Baseline characteristics of the 238 cleaners who were offered participation in the text message survey, and of those, who consented to participate and responded to more (high responders) or less (low responders) than 80% of both questions. * = significant difference between high and low responders (*p *< 0.05).

### Course of low back pain and leisure time physical activity during the 52 weeks

**Figure 3:**
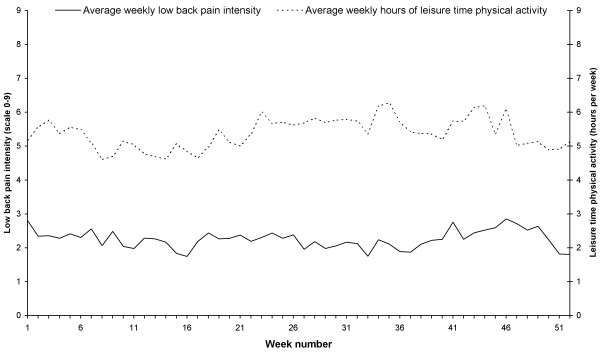
**Weekly mean low back pain intensity (scale from 0 to 9, with 0 as no pain within the last 7 days and 9 as worst pain imaginable) and mean hours of leisure time physical activity (time spent on activities like aerobics, running, bicycling, swimming and active transport) during the previous 7 days for the high responders, (those who responded more than 80%, n = 49)**. Time period was from Week 46 (November) 2008 to Week 45 (November) 2009 and each weekly dot covers 48 to 49 responses.

Figure 3 shows mean hours per week of LTPA and mean weekly LBP intensity each week throughout the 52 weeks among the high responders (n = 49).

The total mean LBP intensity for the high responders throughout the 52 weeks was 2.3 on a 10-point scale, with a standard deviation (SD) between participants of 2.1 and a coefficient of variation (CV) between participants of 91.3%. The highest yearly mean LBP intensity for a high responder was 6.6 (SD 1.9) and the lowest was 0.0.

The total mean hours per week of LTPA for the high responders throughout the 52 weeks was 5.3 with a SD between participants of 3.0 and CV between participants of 56.6%. The highest yearly mean LTPA per week for a high responder was 12.3 hours (SD 1.9) and the lowest was 0.0.

**Figure 4:**
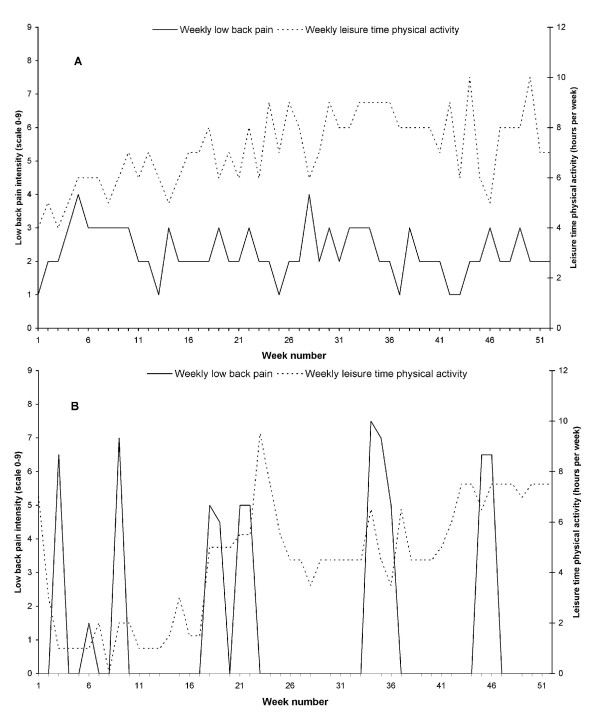
**a) Example of a cleaner who reported constant low to moderate LBP intensity (Black line) throughout the year and the simultaneous LTPA (Grey line), b) Example of a cleaner who reported a larger variation in LBP intensity (Black line) and the simultaneous LTPA (Grey line)**.

Figure 4 shows two examples of the reported weekly LBP intensity and hours per week of LTPA throughout the 52 weeks for (a) a cleaner who reported constant low to moderate LBP intensity throughout the year, and (b) a cleaner who reported a larger variation in LBP intensity, ranging from 0 to 7.5 on the scale from 0 to 9.

### Correlation between leisure time physical activity and low back pain intensity

**Figure 5:**
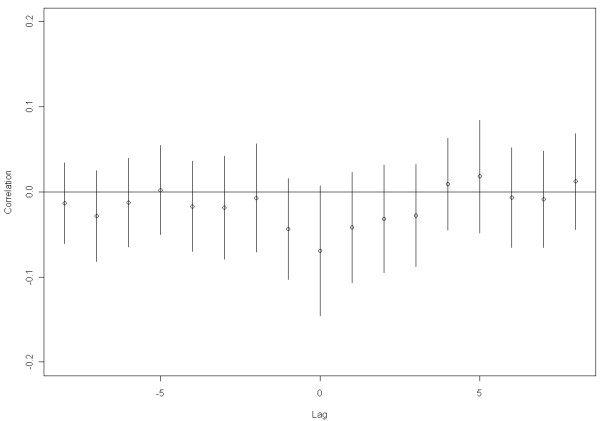
**Average Pearson correlation coefficients with 95% confidence intervals (CI) between hours of leisure time physical activity and low back pain intensity over the 52 weeks for cleaners answering more than 80% of the text messages (n = 49)**. When time lag (week) = 0, the correlation coefficient is between leisure time physical activity and low back pain reported in the same week (e.g. leisure time physical activity in Week 3 vs. low back pain intensity in Week 3). Lag = -1 when leisure time physical activity is correlated with low back pain intensity in the previous week (e.g. leisure time physical activity Week 3 vs. low back pain intensity Week 2).). Lag = +1 when leisure time physical activity is correlated with low back pain intensity in the following week (e.g. leisure time physical activity Week 3 vs. low back pain intensity Week 4).

Figure 5 presents the mean Pearson correlation between LTPA and LBP intensity for the high responders. The correlation between hours of LTPA and LBP intensity in the same week (time lag 0) was negative but numerically relatively low (r = -0.069) and was not statistically significant (*p *= 0.08). When LTPA was correlated with LBP intensity in the previous week (e.g. LTPA in Week 3 vs. LBP intensity in Week 2) or when LTPA was correlated with LBP intensity in the following week (e.g. LTPA in Week 3 vs. LBP intensity in Week 4), non-significant and numerically lower correlations were found.

### Acute low back pain episode and leisure time physical activity

Of the 140 participants who responded to the text message survey, 82 experienced at least one acute LBP episode. The highest number of acute LBP episodes for one participant was seven. In total, 188 acute LBP episodes with text message responses in the 4-week follow-up period were included in the analysis. Initial LBP intensity was 1.55 (SD 1.90) for episodes with maintained hours of LTPA and 1.21 (SD 1.65) for episodes with decreased hours of LTPA.

We found no significant effect of maintained LTPA on the return to initial pain intensity within the 4-week follow-up period after an acute LBP episode. Having episodes with decreased hours of LTPA as a reference, a positive but non-significant effect of maintaining LTPA was estimated through an odds ratio (OR) of 1.02 with 95% confidence interval (CI) of 0.50-2.09 when controlling for intervention group. When controlling for age and gender as well as for intervention group, the estimated OR was 0.94 (CI 0.45-1.95).

The mean increase in LBP intensity in the 4-week follow-up was higher when LTPA was maintained than when LTPA was decreased, although not reaching statistical significance. The mean LBP increase on a 10-point scale was 0.06 (CI -0.42-0.54) higher for maintained compared with decreased LTPA during follow-up when controlling for intervention group. When controlling for age and gender as well as intervention group, the mean LBP increase was 0.09 (CI -0.40-0.58) higher for maintained compared with decreased LTPA during follow-up, but still the difference did not reach statistical significance.

## Discussion

The main findings of the study were that (1) the correlation between LBP intensity and hours of LTPA throughout the 52 weeks was low and non-significant, and (2) maintaining LTPA during an episode of acute LBP did not have a positive effect on LBP in the following 4 weeks.

To our knowledge, this study is the first to investigate LBP and LTPA in an occupationally active population (cleaners) with a high risk of LBP on a weekly basis over 52 weeks. The repeated measures of LBP showed an average intensity of LBP throughout the 52 weeks of 2.3 on a 10-point scale. However, the weekly data on LBP for the 52 weeks show a high inter- and intra-individual variability in this occupationally active population. For example, the average LBP intensity ranged from 0.0 in one cleaner to 6.6 in another cleaner. Moreover, some cleaners had a relatively constant low to moderate LBP throughout the year (for example Figure 4a), while others had a large variation in LBP intensity throughout the year with defined periods of acute LBP lasting for a few weeks (see for example Figure 4b). These findings highlight the importance of repeated data on LBP throughout a longer period of time for valid estimates of the course of LBP in a person. Nevertheless, with the current group of high responders, the mean variation was within 1 point on the 10-point scale and no clear seasonal variation was evident. This finding suggests that in larger intervention studies, differences less than this, should not be considered true changes and instead, simply be viewed as normal variation.

Based on the correlation analyses, no significant associations between LBP intensity and hours of LTPA on a weekly basis throughout the 52 weeks were found. Moreover, the correlation between LBP and LTPA in the previous or succeeding weeks was also very low, indicating no systematic time-dependent relationship between the variables. These findings are consistent with studies on both acute and chronic LBP patients [10,16]. Therefore, LBP intensity and hours of LTPA do not seem to be closely related in this study population of cleaners. However, it should be mentioned that the low level of both LBP and LTPA limits the possibility for identifying such an association. Another point to consider is that the type of LTPA performed by the cleaners may explain the weak correlation; while LTPA is generally believed to benefit LBP, there are also some forms of LTPA such as regular home improvement activities and high intensity sports that may actually increase the risk of LBP [30]. In the present study, the low background level of LTPA may possibly explain the lack of effect of maintaining or lowering LTPA.

Maintaining the usual level of LTPA during an episode of acute LBP was expected to positively influence LBP in a 4-week follow-up period. However, among the 82 cleaners experiencing at least one episode of acute LBP, those maintaining their usual LTPA during an episode of LBP did not benefit from a lower pain level or higher probability of returning to the initial pain level in the following four weeks compared with cleaners decreasing their LTPA during acute LBP. Apart from the already mentioned general low level of both LBP and LTPA, one of the reasons for this finding may be that some types of LTPA reduce, while others increase, the risk of subsequent LBP as shown among acute LBP patients [31]. Another possibility is that among occupationally active workers, maintaining the level of LTPA during an episode of LBP may not be enough to make a difference to the acute LBP intensity. Therefore, more evidence is needed to lend support to the existing clinical guidelines with respect to acute LBP when targeting an occupationally active population.

### Strengths and limitations of the study

An obvious strength of this study is the repeated measures of LBP and LTPA on a weekly basis throughout 52 weeks, enabling investigation of the course of LBP throughout a year, the time-dependent correlation between LBP and LTPA, and the analysis of the effect of maintained LTPA during an episode of acute LBP. Moreover, it is well known that questionnaire based data on 3 or 12 months prevalence and intensity of LBP and LTPA has a high risk of recall bias [19,32-34]. Recall bias is minimised by using the text message survey method, enabling weekly measures of LBP and LTPA with high time resolution. A second strength of the study is the occupationally active population of workers adding to our knowledge, which to date had been based on patients used in most LBP studies. On the other hand, it is a limitation that this method relies on self-reported data not validated with objective measurements of LTPA among workers. Moreover, text messaging only allows short and limited numbers of questions and the validity of a single question from the IPAQ as a measure of the total amount of LTPA it is not known.

Since the background prevalence of LBP is high in the general population, providing a clear definition of new episodes of LBP is not feasible. Instead, a pragmatic approach with a clinically relevant change in pain status was used for defining a new episode of LBP. This may have introduced some bias, since regardless of initial LBP intensity, a new episode of acute LBP was defined as an increase of 2 or more in pain intensity on a 10-point scale. However, the initial LBP intensity was not different between LBP episodes with maintained or decreased LTPA, and therefore this did not introduce such a bias into these comparisons. An increase of 2 on a 10-point scale is about twice the change often considered clinically relevant in intervention studies with the aim of reducing pain [25,26]. In the current population, this increase is also about twice the normal variation over the year.

The study was nested in a clinical trial aimed at reducing musculoskeletal pain and pain-related fear of movement (kinesiophobia). Accordingly, the participants were randomised and participated in different interventions throughout the 52 weeks of measurement. Therefore adjustment for intervention group in the statistical models was performed so that this did not influence the findings of the study.

The response rate in our study was lower than that previously achieved by other research groups and the low response rate may limit the generalisability of the results Other studies using weekly text messages have achieved response rates of more than 60% after 18 weeks [17,18]. This may be due to a more motivated study population of patients with LBP in previous studies compared with the occupationally active population in this study. The nested design of the study allowed us to analyse for possible selective reporting. Indeed, the participants in the high responder group were older, more were ethnic Danes and they had longer job seniority as cleaners. However, high and low responders did not differ in LBP and LTPA.

## Conclusions

The current findings indicate that hours of LTPA and intensity of LBP are not closely correlated on a weekly basis throughout a year, and that maintaining LTPA during an episode of acute LBP does not have a positive effect on LBP in the following 4 weeks. It may therefore be questioned if the clinical recommendations for acute LBP also apply to workers with high physical work demands, although this was not specifically tested in the present study. Future studies evaluating the effect of advice to stay active in populations who have high physical work demands are needed. The generalisability of the current study is unknown. However the findings illustrate the need for documentation of relevant and specific clinical guidelines with respect to acute LBP for different populations, such as patients and workers in occupations with different job characteristics. Prospective studies with repetitive measurements, including objective measures of physical activity, are lacking and clearly needed.

## Competing interests

The authors declare that they have no competing interests.

## Authors' contributions

MBJ, AHO, and KS, were involved in the concept and design of the study. TJ was responsible for the practical part of the study, data sampling and processing. TJ and JVH were responsible for the statistical analysis. All authors were involved in data interpretation. TJ wrote the first draft, and all authors read and approved the final manuscript.

## Pre-publication history

The pre-publication history for this paper can be accessed here:

http://www.biomedcentral.com/1471-2474/13/28/prepub
